# Metabolic Symbiosis and Immunomodulation: How Tumor Cell-Derived Lactate May Disturb Innate and Adaptive Immune Responses

**DOI:** 10.3389/fonc.2018.00081

**Published:** 2018-03-23

**Authors:** Alexandre Morrot, Leonardo Marques da Fonseca, Eduardo J. Salustiano, Luciana Boffoni Gentile, Luciana Conde, Alessandra Almeida Filardy, Tatiany Nunes Franklim, Kelli Monteiro da Costa, Celio Geraldo Freire-de-Lima, Leonardo Freire-de-Lima

**Affiliations:** ^1^Faculdade de Medicina, Universidade Federal do Rio de Janeiro, Rio de Janeiro, Brazil; ^2^Laboratório de Imunoparasitologia, Instituto Oswaldo Cruz, Fundação Oswaldo Cruz (Fiocruz), Rio de Janeiro, Brazil; ^3^Instituto de Biofísica Carlos Chagas Filho, Universidade Federal do Rio de Janeiro, Rio de Janeiro, Brazil; ^4^Instituto de Microbiologia, Departamento de Imunologia, Universidade Federal do Rio de Janeiro, Rio de Janeiro, Brazil

**Keywords:** cancer, metabolism, lactate, immune evasion, cytokines

## Abstract

The tumor microenvironment (TME) is composed by cellular and non-cellular components. Examples include the following: (i) bone marrow-derived inflammatory cells, (ii) fibroblasts, (iii) blood vessels, (iv) immune cells, and (v) extracellular matrix components. In most cases, this combination of components may result in an inhospitable environment, in which a significant retrenchment in nutrients and oxygen considerably disturbs cell metabolism. Cancer cells are characterized by an enhanced uptake and utilization of glucose, a phenomenon described by Otto Warburg over 90 years ago. One of the main products of this reprogrammed cell metabolism is lactate. “Lactagenic” or lactate-producing cancer cells are characterized by their immunomodulatory properties, since lactate, the end product of the aerobic glycolysis, besides acting as an inducer of cellular signaling phenomena to influence cellular fate, might also play a role as an immunosuppressive metabolite. Over the last 10 years, it has been well accepted that in the TME, the lactate secreted by transformed cells is able to compromise the function and/or assembly of an effective immune response against tumors. Herein, we will discuss recent advances regarding the deleterious effect of high concentrations of lactate on the tumor-infiltrating immune cells, which might characterize an innovative way of understanding the tumor-immune privilege.

## Introduction

### Cancer as a Metabolic Disease

Historically, cancer has been considered a product of multiple pathologies. In second century AD, the philosopher and physician Claudius Galenus was the first to employ the Greek word *onco* (swelling) for all types of tumors, leaving Hippocrates’ term *karkinos* exclusively for malignant tumors. During his time, Galenus asserted that tumors were the result of “black bile” accumulation. It was only during the nineteenth century that this theory was revisited and cancer begun to be perceived as the result of acquired metabolic abnormalities (1). Nowadays, it is well accepted that cancer development and progression is modulated by the disordered growth of cells featuring self-sufficiency of growth signals, evasion of apoptosis, angiogenesis, invasiveness, and metastasis (2, 3). When cells break free from the restraints on cell division, they start assuming inappropriate proliferation rates and distinct metabolic profiles, becoming abnormal in their own way (4, 5). Cells originating from solid tumors may gain the ability to dissolve the extracellular matrix (ECM), invade nearby tissues, reaching the bloodstream or lymphatic vessels, or remain within the boundaries of the original tissue, being characterized as malignant or benign tumors, respectively. Several genomic changes lead normal cells through malignant transformation. These changes can be anything from point mutations and deletions to chromosome rearrangements, as long as they result in irreversible changes affecting cell cycle (6). Any individual suffers several mutations in various cell types during its lifetime, due to diverse exogenous or endogenous factors. Most of these mutations are promptly corrected or lead to apoptosis. The accumulation of uncorrected mutations leads to the development of benign or malignant tumors (3). Loss of tumor suppressor factors, germ-line mutations, and overexpression of oncogenes are some of the changes that may collaborate for the occurrence of somatic mutations that escape DNA repair processes (7, 8).

The tumor microenvironment (TME) comprises both cellular and non-cellular components (9–11). The acellular components include the ECM, as well as soluble signals secreted by transformed and tumor-associated cancer cells. Several cell types associate with tumors, including fibroblasts, endothelial cells, and immune cells. Together, all components form an organ-like structure capable of interacting with the organism as a whole (12–14). To maintain tumor growth, several adaptations may be driven by neoplastic cells. A well-known mechanism is the formation of immature and abnormal vessels, in a phenomenon named neoangiogenesis (15, 16). In this context, both the platelet-derived growth factor and the vascular endothelial growth factor (VEGF) are recognized as the main proangiogenic signals upregulated by cancer cells during tumor growth (15, 17, 18).

The incredible proliferation rate of tumor cells can make a single mutated cell generate a tumor of ≈1 cm in diameter containing over 10^9^ cells. Such a high proliferation ratio demands effective metabolic pathways, capable of meeting the steep energy requirements while supplying the biosynthetic precursors that maintain cell anabolism and redox balance in the neoplastic cell (19). Reprogramming of cellular metabolism has been observed in several types of cancer and is considered a hallmark of this disease (3, 20). The elucidation of these atypical metabolic activities is a lively field in the study of cancer biology, showing great potential for the development of novel therapeutic approaches. Several studies have shown that inhibition of some metabolic pathways of cancer cells is able to prevent tumor growth and metastasis (21, 22).

## Metabolic Symbiosis: A Proposed Concept of Energy Management Between Cancer Cells in the TME

**Figure 1:**
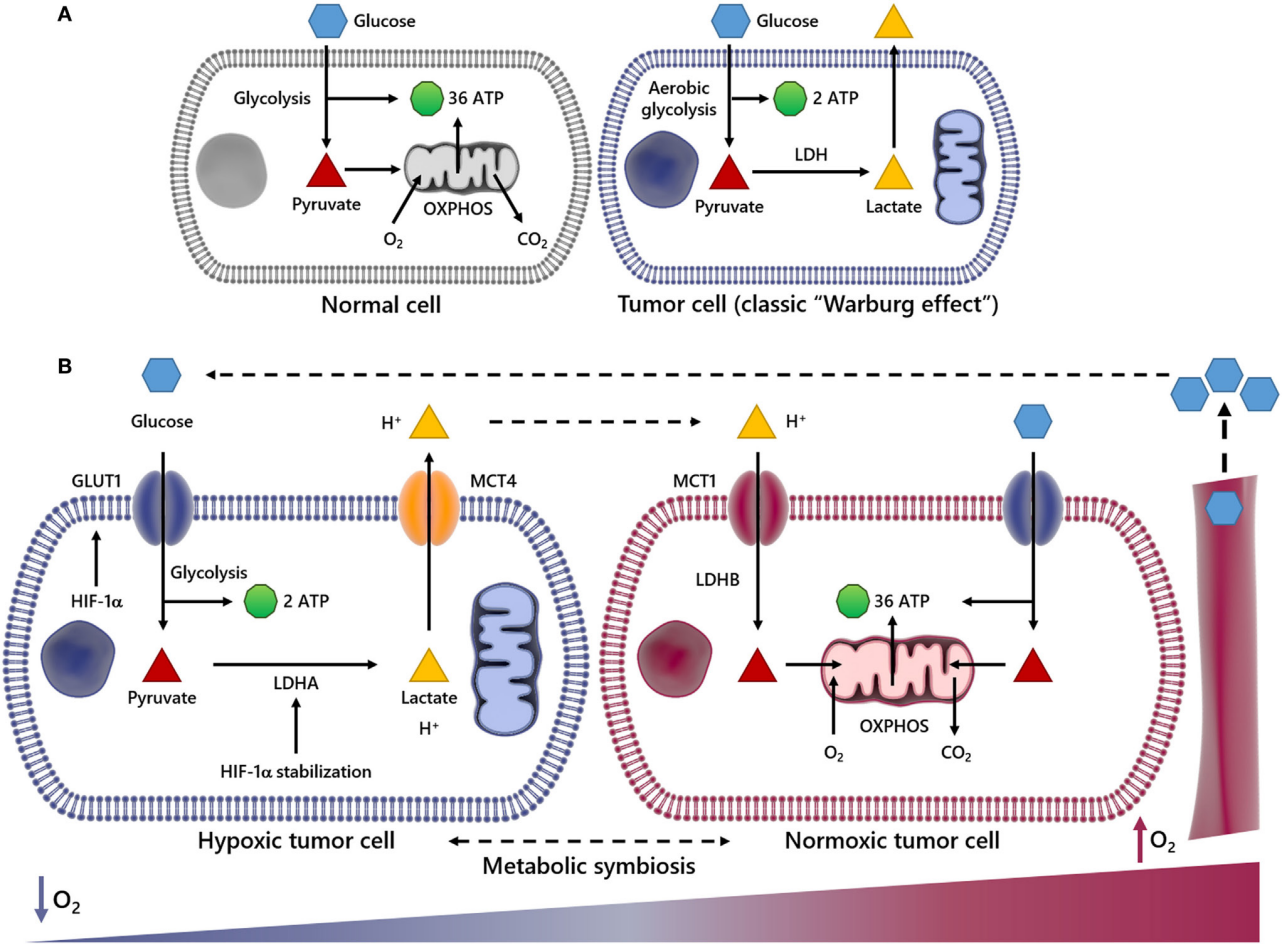
Scheme summarizing metabolic differences between normal and cancer cells and metabolic symbiosis. In normal cells, glucose (Glc) is initially metabolized to pyruvate and further to carbon dioxide (CO_2_) through tricarboxylic acid cycle and oxidative phosphorylation (OXPHOS) processes in the mitochondria, generating 36 ATP molecules per Glc molecule consumed **(A)**. In this process, O_2_ is indispensable, since it is used as the final electron acceptor **(A)**. However, in cancer cells undergoing aerobic glycolysis (Warburg effect), Glc is broken down into pyruvate and finally converted into lactate, deviating Glc metabolites from energy production to anabolic process. This event generates two ATP molecules per Glc molecule. The panel **(B)** illustrates an event named metabolic symbiosis. It has been well documented that when cancer cells are near or distant of blood vessels, they are well or poorly oxygenated, respectively. It is also known that when cancer cells are subject to low oxygen tension (↓O_2_) hypoxia-inducible factor-1α (HIF-1α) is stabilized, increasing the transcriptional activation of genes encoding glucose transporters (GLUTs), lactate dehydrogenase A (LDHA), as well as the uptake of Glc and secretion of lactate and protons out of cytoplasm through the monocarboxylated transporter 4 (MCT4). However, when transformed cells are close to blood vessels and the availability of O_2_ is enough, lactate is taken by monocarboxylated transporter 1 (MCT1) and utilized as energy source after conversion into pyruvate by lactate dehydrogenase B (LDHB). In this way, lactate may not be pointed out as a waste metabolite, since it is reused by different cell subpopulations in a tumor.

The impact of the acidosis induced by lactate and protons in the TME is a hot topic in cancer research (23–25). It is a well-established fact that a high enough lactate production can overcome the cellular proton buffering capability, resulting in a decrease of the cellular pH. Such condition, besides influencing the dynamics of waste and reuse of energy by cancer cells, modulates the function of distinct tumor-associated cell types as well (26–29). Several papers published over the last 10 years, demonstrated that when cancer cells experience low tension of oxygen, the hypoxia-inducible factor-1α (HIF-1α) transcription factor is stabilized, increasing glucose (Glc) uptake and secretion of substantial levels of lactate and protons out of cytoplasm by the monocarboxylated transporter 4 (MCT4) (Figure 1B), promoting a biochemical event termed lactic acidosis. By contrast, when cancer cells are adjacent to blood vessels and oxygen availability is sufficient, the transformed cells preferably use lactate as energy source (29–33). For this reason, lactate should not be considered a waste metabolite. In fact, it is reused by different cell subpopulations in a tumor (28, 29). Recently, Lee and colleagues (34) showed that an oxygen-regulated protein (NDGR3), which is usually degraded under normoxia *via* the prolyl hydroxylase 2/Von Hippel–Lindau (PDH2/VHL) system, becomes protected from degradation when bound to lactate. The authors demonstrated that when stable, NDRG3 is able to bind the proto-oncogene c-Raf, a serine/threonine-protein kinase, and induce activation of the Raf–ERK pathway, thus promoting cell growth and angiogenesis. However, inhibition of lactate production compromises NDRG3-mediated hypoxia responses (34).

This metabolic model of lactate reuse in the TME has been described as a metabolic symbiosis, where lactate works as a medium to convey energy from highly glycolytic/hypoxic transformed cells to more oxidative cancer cells (35, 36). In the TME, the uptake of lactate and protons by oxygenated cancer cells occurs in a dynamic way through the monocarboxylated transporter 1 (MCT1) (Figure 1B), which has been previously identified as gatekeeper of this metabolic symbiosis (29). In the same study, the authors demonstrated that cells with inhibited or silenced MCT1 became more sensitive to cell death, which may support lactate management within the TME as a valid therapeutic strategy. Therefore, it would plausible to speculate that the high-lactate concentration at the intercellular space might affect the functionality of diverse tumor-associated cells, including those of the innate and adaptive immune system (see sections below).

## The Effect of Lactate in the TME

In normal cell metabolism, the consumed Glc is catabolized into pyruvate, which is then transported to the mitochondria to fuel the tricarboxylic acid cycle in a series of redox reactions. The resulting free electrons go through the electron transport chain (ETC), beginning the oxidative phosphorylation (OXPHOS) and ultimately leading to a high production of ATP (37) (Figure 1A). In the early 1920s, Otto Warburg observed that tumor cells remain in glycolytic state, constitutively absorbing Glc and converting pyruvate to lactate, in the presence of oxygen (Figure 1A). Lactate production is 40-fold greater in tumor cells, so the transport of lactate to the ECM by MCTs (38–41) is essential for the glycolytic switch. This metabolic behavior is named “Warburg Effect,” or aerobic glycolysis, one of the main characteristics studied in cancer metabolism (19) (Figure 1B). Glycolysis produces ATP faster yet less efficiently than OXPHOS, forcing the tumor cell to consume much more Glc than a normal cell to produce enough energy to maintain its high proliferative status. Therefore, the glycolysis is an advantage for the tumor cell only when Glc supply is not limited. The importance of Glc for the metabolism of cancer cells is so evident that low-carbohydrate diet as a therapeutic approach for cancer patients, aiming to starve tumor cells, was described to limit the growth of incurable cancers in a pilot trial with 10 patients (42). In that regard, the uptake of a radioactive Glc analog, [^18^F]fluorodeoxyGlc, is used as a diagnostic tool for the positron emission tomography (FDG-PET) imaging of highly proliferative tumor regions (43, 44). Currently we know that tumor cells primarily fulfill their energetic needs by the oxidation of Glc, glutamine and other nutrients coupled to the ETC, using oxygen as the final acceptor of electrons (45, 46). In cancer cells, the anaerobic respiration is optional, and there is no mitochondrial defect (47); in fact, tumor cells still retain OXPHOS and mitochondrial activity (39). The reduced mitochondrial activity is a direct result either of oxygen deprivation or activation of HIF-1α (48, 49), which is able to promote the transcriptional activation of genes encoding glucose transporters (GLUTs), as well as glycolytic enzymes, such as lactate dehydrogenase A (LDHA) (50). When the supply of oxygen is low, LDHA is essential to sustain glycolysis and the production of ATP by regenerating NAD^+^ form NADH. In this way, HIF-1α regulates the production of lactate, the end by-product of this reaction, which consumes two ATP but generates four ATP, generating two net ATP per Glc molecule as seen in (Figures 1A,B) (50). Upstream of HIF-1α and the previously discussed Raf–ERK, the Ras oncogenic pathway seems to be critical for the metabolic reprogramming observed during carcinogenesis. Overexpression of oncogenic H-Ras^V12^ was able to drive immortalized fibroblasts to consume more Glc and to release more lactate (51). Conversely, Ras inhibition in a model of glioblastoma (GBM) effectively shuts down Glc uptake and glycolysis itself, leading to the downregulation of 12 genes from the glycolytic pathway and increased extracellular pH due to reduced lactate efflux (52). The role of PDH2 is prominent in this, since oncogenic H-Ras signaling, as well as hypoxia, triggers oxidative stress, PDH2 dimerization, and inactivation, leading to HIF-1α stabilization and ultimately the OXPHOS to glycolysis shift (53).

**Figure 2:**
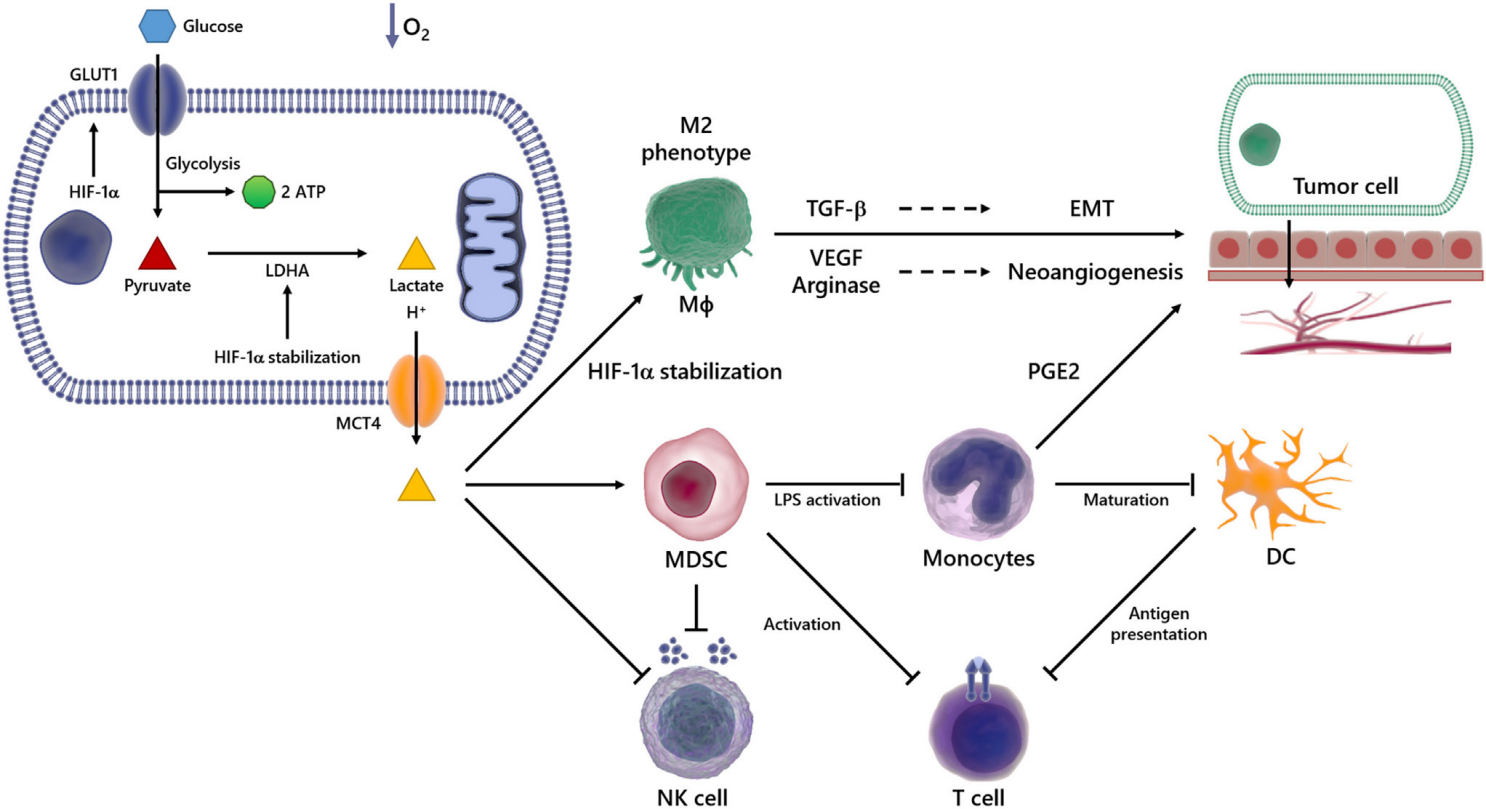
Overview of immunosuppressive effects of lactate in the tumor microenvironment (TME). In a hypoxic environment, Glc enters the cell *via* glucose transporter (GLUT) 1 and is broken down into pyruvate and then in lactate, which is transported out of the cell *via* monocarboxylated transporter 4 (MCT4). The lactate produced by transformed cells culminates in an acidified TME. This phenomenon is able to suppress the anticancer immune responses, particularly through impaired T and natural killer (NK) cells activation, reduced antigen presentation, compromised dendritic cell (DC) differentiation and maturation. It also promotes the emergence of the M2 Mϕ, which secretes high levels of pro-carcinogenic cytokines, such as transforming growth factor-beta (TGF-β) and vascular endothelial growth factor (VEGF), involved in processes such as epithelial–mesenchymal transition (EMT) and angiogenesis, events implicated in metastasis and cancer progression.

An immediate consequence of the Warburg effect is the accumulation of lactate and protons in the TME (23, 54). It has been shown that in patients diagnosed with different stages of cervical cancer, primary tumors exhibiting high-lactate levels often lead to the manifestation of metastatic foci (55). The same group, using human larynx squamous carcinoma cells, showed that increased lactate concentration can augment cell motility and migration, corroborating the data observed in patients (56). In addition, the use of siRNA to inhibit the expression of LDHA, whose expression can be induced by lactate itself, is able to inhibit the migration of glioma cells as well as downregulate active matrix metalloproteinase-2 (57). Increased lactic acid is also responsible for the overexpression of factors related to tumor progression, such as CD44, hyaluronic acid and transforming growth factor-beta (TGF-β) (58) (Figure 2), a pro-carcinogenic cytokine able to activate the epithelial–mesenchymal transition process, an event that permits dissemination of tumor cells from the primary site into the surrounding stroma, setting the stage for metastatic spread (59–61). In addition, due to its antioxidant properties, increased lactate concentrations may offer a degree or resistance against any therapy relying on the production of oxygen reactive species, such as radiotherapy (62). As it stands, further studies on the production of lactate by solid tumors represent an important step toward the understanding of tumor progression and malignancy, as well as for therapy development.

The TME is characterized by acidity and low oxygen tension (63, 64), events capable of modulating not only the growth and survival of tumor cells but also the recruitment of inflammatory cells that are reeducated in the microenvironment to favor tumor spread and metastasis. In this scenario, various inflammatory cells, such as T lymphocytes, dendritic cells (DCs), natural killer (NK) cells, and macrophages (Mϕ), acquire pro-carcinogenic properties (Figure 2) (65–69). Recruitment and accumulation of those cells in the TME is an essential phenomenon to sustain the tumor growth (70). The immune system’s role in the first phases of tumor establishment is well described, being capable of detecting and destroying cancer cells, halting their growth and spread, in a phenomenon termed immunosurveillance (71, 72), which was initially proposed by Paul Ehrlich and later developed by Sir Frank Macfarlane Burnet and Lewis Thomas (13, 73). Defects in this event might favor tumor progression and, consequently, the acquisition of a malignant phenotype. Any cancer cell that manages to escape death triggered by immune response could still have their proliferation hindered by immune mechanisms, reaching an equilibrium. On the other hand, the immunogenicity is molded through selective pressure exerted by the immune system, in an event termed immunoediting (74–77). Consequently, novel tumor variants emerge, bearing more mutations, making them more likely to evade detection and elimination by immune effector cells like NK and CD8^+^ T cells (74, 78). The immunoediting stage is the longest phase, and it is characterized by the dynamic coevolution of cancer and immune cells (78–80). Ultimately, cells reach an escape phase, where the accumulation of edited cells drives tumor growth and the manifestation of clinical symptoms (74). The presence of the immune system in the TME undoubtedly compromises tumor growth and, in fact, correlates with favorable prognosis in some cancer types, such as renal, ovarian, colorectal, and breast cancers (74, 81). The expression of molecules able to compromise cell-to-cell interaction (3, 82–84), as well as soluble factors such as VEGF (85), cytokines (86–88), prostaglandin E2 (PGE2) (89), soluble Fas and FasL (90), and soluble MICA (91), all contribute to the appearance of multifaceted local and regional immunosuppressive networks (92–94). For example, the occurrence of the IL-4, TGF-β, IL-13, and IL-10 cytokines in the TME induces the emergence of M2 instead of M1 Mϕ (86, 87). In addition, secretion of nitric oxide, IL-10, arginase-1, IL-6, and VEGF promotes cell death and avoids the antitumor function of immune cells (95–99).

As stated earlier, the TME is rich in lactate (38–41), an immunosuppressive soluble factor that promotes cancer development (54, 74, 100). Particularly, several studies have shown that tumor-derived lactate is capable of inhibiting the activation of immune cells such as monocytes, Mϕ and T lymphocytes (101–103). It has been demonstrated that high LDHA levels are deeply correlated with tumor size, as well as with the clinical stages of the disease (104, 105). Accordingly, the infiltration of immune cells in the TME correlates with high LDHA expression and/or activity (106). Besides lactate accumulation in the primary tumor site, its immunosuppressive properties can outspread to distant sites, thus stimulating invasion and metastasis in a paracrine fashion (107) (Figure 2).

Despite being mainly produced by skeletal muscle, various tissues generate lactate, and its elimination in healthy conditions is handled primarily through the liver and secondarily through the kidneys (108). The citric acid cycle is also a source of lactate, as pyruvate can be diverted to lactate and NAD^+^ generation through LDH activity (109, 110). The high amounts of lactate in the extracellular microenvironment contribute to lowering the extracellular pH, which can be as low as 6.0–6.5 (111), producing acidosis and inducing angiogenesis and a reduction in efficacy of the immune system (101, 112, 113). Tumor-associated immune cells from myeloid and lymphoid origin can be modulated by hypoxic conditions as well as high levels of lactate, then favoring the acquisition of malignant phenotypes (23, 64, 114–116).

## Lactate and Myeloid Cells

Over the last 15 years, several studies demonstrated that tumor-derived lactate modulates the functionality of immune cells, contributing to the establishment of an immunosuppressive microenvironment, which favors the developing of tumors (106, 117–119). Lactate promotes the development of myeloid-derived suppressor cells (MDSCs), the most prominent bone marrow-derived cell population that exerts broadly immunosuppressive functions (106). In the TME, MDSCs potently suppress both innate and adaptive immunity by preventing the maturation of DCs, suppressing NK-cell cytotoxicity, inhibiting T-cell activation, and favoring the differentiation of regulatory T cells (Figure 2) (117).

In addition, lactate suppresses monocytes’ LPS-induced activation by influencing their gene expression. Particularly, the expression of most LPS-induced genes was significantly delayed in the presence of lactate, including TNF, IL-23, CCL2, and CCL17. These effects are mediated by delayed LPS-induced phosphorylation of protein kinase B (AKT) and degradation of IkB, with reduced nuclear accumulation of NFκB (119). Furthermore, lactate stabilizes the transcription factor HIF-1α in monocytes, which ultimately promote the expression of PGE2 and the growth of human colon cancer HCT116 cells (120).

Another suppressive function of lactate is to impair the differentiation of monocytes into Mϕ or DCs in the TME (118, 121–123) and in non-tumor conditions (103, 124) (Figure 2). It was reported that lactate blocks LPS activation of bone marrow-derived Mϕ (BMDMs) (123), and also, in hypoxia or normoxia, lactate drives tumor-associated Mϕ polarization to the “tumor friendly” M2 profile (Figure 2) (125, 126). Mechanistically, lactate stabilizes HIF-1α, which leads to the transcription of a broader set of M2-associated genes, including VEGF, TGF-β and arginase-1, as well as Fizz1, Mgl1, and Mgl2 (Figure 2) (98, 125–128). The role of PDH2 as a regulator of the metabolic reprogramming in Mϕ was observed in both RAW264 cells and in primary BMDM, since transfection with shRNA targeting PDH2 or conditional PDH2 knocking out led to decreased ATP levels along and increased lactate release into the medium (129). M2 Mϕ and their products favor tumor growth and metastasis by suppressing antitumor immune responses, activating and enhancing angiogenesis. Particularly, VEGF triggers the development of neovascularization of the tumor. Similarly, arginase-1 plays an indirect role in angiogenesis through reorganization of the tumor ECM and contributes for the generation of essential metabolites during cell division, such as polyamines, supporting cancer cells growth (130–133). The importance of arginase-1 in tumor development was demonstrated by the use of arginase-1 KO mice, which presented tumors 50% smaller than tumors from wild type mice (125, 134). Finally, distinct studies have shown that when present in high levels, lactate inhibits antigen presentation and IL-12 production by DCs (54, 135) (Figure 2) and enhances IL-10 production as well, generating an immunosuppressive profile in the TME (136, 137).

## Lactate and Lymphoid Cells

The immunobiological effects of lactate on immune cells from lymphoid origin have been mainly investigated in NK and T cells. The cytotoxic effect mediated by both cell types is of fundamental importance in immunological surveillance against the emergence and spread of malignant disease. NK cells represent large granular lymphocytes that induce their antitumor responses through the ligation of particular cell-surface receptors (138), such as the natural killer group 2, member D receptor (NKG2D), which induces the release of cytotoxic granules that promote the lysis of cancer cells (139, 140).

An elegant study developed by Husain and colleagues (106) revealed that the low production of lactate in LDHA-depleted tumors was able to improve the cytolytic functions of NK cells. However, when NK cells where pretreated with lactate *in vitro*, its cytolytic property was compromised and/or abrogated. The molecular mechanism responsible for such effect was investigated, and the authors demonstrated that the decline of NK cytotoxic activity was promoted by the lower expression of granzyme and perforin in lactate-treated cells (106). Furthermore, it was described that lactate works as a potent inhibitor of histone deacetylases, suggesting that lactate might be able to regulate (at transcriptional level) several genes involved not only in cell metabolism but also in immune responses, such as NCR1/NKp46, an activating NK cell receptor (141, 142).

In 2014, Crane and coworkers demonstrated that GBM cells secrete LDH-5, an enzymatically active isoform of LDH (143), that besides catalyzing the conversion of pyruvate to lactate in an efficient way (144), is also capable to upregulate HIF-related pathways (145) and induce the expression of NKG2D ligands on healthy monocytes, thus subverting antitumor immune responses (143). In a previous study realized by the same group, it was demonstrated that TGF-β downregulates NKG2D expression in NK cells *in vitro* (146), supporting the idea that the elevated production/concentration of TGF-β in acidic TME is one of the main evasion mechanisms adopted by cancer cells (146).

It is well accepted that a robust presence of T cells in the TME is associated with good clinical outcome in distinct types of cancers (147–149). It is important to notice that new progresses in cancer immunotherapy are allied to the use of monoclonal antibodies directed against T cell-immune checkpoints. Examples include CTLA4 (149–151) and PD-1 (152, 153). These outstanding findings undoubtedly confirm the necessity of an effective T cell activation to control tumor growth and spread (147). As with other types of immune cells, cancer cells limit T cell immunity by distinct ways. In this regard, the acidification of the TME is a clear example, and several papers have demonstrated that lactate plays a pivotal role in this process (54, 154–156). As with transformed cells, activated T cells may generate energy through aerobic glycolysis (157–159). The upregulation of glycolytic enzymes intensifies the uptake of Glc and glycolytic rate, favoring the secretion of lactate into the microenvironment (157). It is possible to imagine that when together in the TME, both cancer cells and activated T lymphocytes significantly increase the production/secretion of lactate. As it stands, it has been demonstrated that the very acidic microenvironment suppresses the proliferation and cytokine production by activated T cells (101). A recent study developed by Brand and colleagues revealed that pathophysiological concentrations of lactic acid repeal the upregulation of the nuclear factor of activated T cells in both NK and T cells, which significantly reduced the production of IFN-γ (160). These results corroborate previous findings that lactic acid is able to downmodulate the function of cells from lymphoid origin, then contributing to tumor escape from immune attack.

## Concluding Remarks and Perspectives

This review presents a snapshot of metabolic changes in cancer cells, describing how, even in aerobic conditions, transformed cells opt for glycolysis instead of OXPHOS to sustain their energy demand, high proliferation and biosynthesis rates, a process named “Warburg effect.” This metabolic reprogramming culminates in a high-lactate and protons output, which is also exported to the extracellular environment by MCT4, generating acidosis, neoangiogenesis, and immunosuppression, directly modulating the TME. Although several genetic, biochemical, and pathophysiologic mechanisms have been identified as causes of malignancy in high-lactate tumors, it remains unclear why seemingly identical tumors may exhibit extreme differences in their lactate levels. Although it is certainly another challenge for future research in this field, several reports point out that high-lactate amounts help in generating a hostile microenvironment for normal cells, affecting the activation and differentiation of effector immune cells as well as antigen presentation and the production of cytokines. Future studies, particularly in solid tumors characterized by highly acidic environments, are needed to better understand the effect of lactate and other “waste” metabolites on cancer progression. The participation of lactate in TME and its immunosuppressive actions not only make it crucial for tumor survival and growth but also turns it into an interesting and promising candidate to therapeutic target in cancer chemotherapy. Following this reasoning, classic and novel drugs that modulate TME pH might be useful as potential immunomodulatory tools in cancer patients, particularly in combination with immunotherapeutic strategies.

## Author Contributions

Wrote the paper: AM, LF, LG, LC, AF, TF, KC, ES, CF-d-L, and LF-d-L. All the authors read and approved the final version of the manuscript.

## Conflict of Interest Statement

The authors declare that the research was conducted in the absence of any commercial or financial relationships that could be construed as a potential conflict of interest.
